# Understanding the challenges of medicine optimisation among older people (aged 60 years and above) from ethnic minority communities with polypharmacy in primary care: a realist review protocol

**DOI:** 10.1186/s13643-025-02920-1

**Published:** 2025-08-15

**Authors:** Nesrein Hamed, Clare Bates, Muhammed Umair Khan, Ian Maidment

**Affiliations:** 1 Aston University, Birmingham, United Kingdom; 2 National Institute for Health and Care Research, London, United Kingdom; 3 University of Birmingham, Birmingham, United Kingdom

**Keywords:** Multimorbidity, Person-centred care, Medication review, Deprescribing, Cultural competence, Health disparities, Medication management

## Abstract

**Background:**

Across many countries, the number of older people from ethnic minority communities is growing due to ageing populations and migration trends. In England and Wales, the population of older people from ethnic minority communities, particularly those aged 60 and above, is also increasing. This demographic change, often accompanied by the prevalence of polypharmacy in these communities, presents unique challenges in the context of medicine optimisation. Failure in this context can lead to exacerbated health disparities, non-adherence, and inappropriate prescribing (whether over or under).

Building on the MEMORABLE study exploring medication management in older people, this review aims to understand the complexities of medicine optimisation, exploring what works and does not work, when and under what circumstances for older people from ethnic minority communities. Key possible areas include cultural backgrounds, traditional beliefs, and systemic barriers that may influence medicine optimisation.

**Methods:**

The review will follow the five-step realist approach that firstly establishes initial programme theories to highlight the expected context, mechanisms, and outcomes. Then a formal search for evidence will be conducted. The third step involves the selection and appraisal of studies screened by title, abstract/keywords and full text based on exclusion/inclusion criteria. Then data from these studies will be extracted, recorded, and coded. The final step will synthesise this information, to test, refine, and expand our initial programme theories and generate context-mechanism-outcome configurations to better understand medicine optimisation in these communities.

**Discussion:**

This review will be conducted in line with the RAMESES reporting standards. By explaining what works, for whom, and in what contexts, the review will generate theory-informed insights into MO for older people from ethnic minority communities with polypharmacy in primary care. These findings can support the development of culturally responsive, person-centred interventions. Results will be shared through peer-reviewed publications and presentations at relevant national and international conferences.

**Trial registration:**

Systematic review registration:

PROSPERO CRD42023432204.

**Supplementary Information:**

The online version contains supplementary material available at 10.1186/s13643-025-02920-1.

## Background

Globally, more people from ethnic minority communities (EMCs) are living longer and entering later life with complex health needs [1]. This pattern is also emerging in the UK, where there has been a significant increase in the number of older people aged 60 years and above from EMCs living in England and Wales [2]. The 2021 Census report found that the population aged 65 years and over was more ethnically diverse in 2021 than in 2011, with an increase from 4.5 to 6.4% in older people from EMCs [3]. This ageing trend presents crucial challenges for these communities in the UK.

Previously, the term ‘BAME’(Black, Asian, and Minority Ethnic) was used to categorise these populations, including groups such as Arab, Jewish, Gipsy, Roma, and Traveller communities [4]. However, the 'Commission on Race and Ethnic Disparities’ (2021) criticised the term for oversimplifying the diversity within and between ethnic groups [5]. As a result, more specific terms are now encouraged, such as specifying particular ethnic groups by name or using terms like ethnic minorities [4, 5]. In this review, the focus will be on a diverse range of ethnicities, including individuals of African, Caribbean, South Asian (Indian, Pakistani, Bangladeshi, Sri Lankan), Middle Eastern origins and individuals of mixed race. The religious diversity of these communities will also be considered. These populations are among the largest ethnic communities in the UK and were chosen to capture a wide range of experiences, influenced by diverse cultures and beliefs. While the white ethnic group remains the majority at 81.7% of the population in England and Wales, the Asian ethnic groups form the second largest category, making up 9.3% of the population, followed by Black ethnic groups at 4.0%, mixed ethnic groups at 2.9%, and other ethnic groups at 2.1% [6].

As people get older, they are more likely to develop simultaneous multiple long-term conditions, as recognised in the NIHR Strategic Framework for Multiple Long-Term Conditions [7]. As a result, the use of five or more medication items (so-called polypharmacy) becomes more common and is associated with multiple risks [8]. These include iatrogenic harm, a higher likelihood of falls, adverse drug reactions, prolonged hospital stays, and readmissions [9–11]. In the UK, issues related to polypharmacy cause 5–8% of unexpected hospital visits, costing the National Health Service (NHS) £530 million and leading to 5700 deaths each year [12]. The detrimental effects of polypharmacy are particularly challenging among older people due to the physiological changes associated with ageing, such as impairment of metabolism and drug excretion, which could further induce drug-drug or drug-disease interactions [13]. In addition, cognitive impairment, which is more common in later life, can further complicate and increase the risk of medication errors for some individuals in this group [14].

Older people from EMCs are disproportionately affected by polypharmacy [15]. While polypharmacy is often linked to ageing and multiple long-term conditions, this does not fully explain the specific and additional challenges faced by older people from EMCs. These factors will be further examined in the following sections.

### Medicine optimisation

The National Institute for Health and Care Excellence (NICE) defines medicines optimisation (MO) as a ‘person-centred approach to safe and effective medicines use, to ensure people obtain the best possible outcomes from their medicines.’ [16]. The strategies and interventions of MO include conducting medication reviews, deprescribing when necessary, performing medicine reconciliations, identifying potentially inappropriate prescriptions, and providing social support [17, 18]. The failure to effectively optimise medicines within these contexts can lead to exacerbated health disparities, non-adherence, and inappropriate prescribing (whether over- or under-prescribing) [19]. These challenges stem from a range of interconnected cultural, individual, and systemic factors that must be considered in MO [20].

Older people from EMCs often bring a richness of traditional beliefs, past healthcare experiences, and potentially even systemic biases they have encountered [21]. Such contexts can profoundly influence their interactions with MO [22, 23]. What may seem like the best treatment from a clinical perspective might not align with someone’s personal or cultural views. For example, some individuals may prefer herbal or spiritual remedies over-prescribed medications [23]. This can lead to non-adherence, as individuals may prioritise longstanding practices over prescribed treatments [23].

Communication is another major issue, as language barriers and cultural differences can make it harder to understand or explain prescribed medications [24]. Health literacy is not just about reading a label, it is about understanding side effects, long-term risks, and making informed decisions [25–27]. So advice often needs to be explained not only in someone’s language, but in a way that also fits their cultural understanding [28, 29]. What seems straightforward in one culture may mean something different in another [29], especially when traditional beliefs about health and illness vary from mainstream medical views [24].

Cultural competence is essential in MO, as it helps practitioners understand and respect patients’ beliefs, preferences, and health practices [28]. Without it, communication can break down, trust may be weakened, and care may be poorly aligned with patient values [28]. This is particularly important during interactions between patients and practitioners in the decision-making process [30–32]. However, many practitioners receive limited training in this area and often face systemic pressures, such as short consultations and heavy workloads, that make achieving MO more challenging [33–35].

### Rationale for this review

The existing research on MO for older people in EMCs is limited and highlights a significant gap in our understanding of how MO works for this population. While some studies have touched on specific challenges like communication barriers and social influence, few have holistically addressed the context of individual, familial, cultural, and systemic factors [36].

The MEMORABLE study, which employed a realist synthesis approach, identified key medication-related challenges among older people, including poor communication and limited recognition of the patient's perspective in medication reviews [20, 37]. While some participants were from EMCs, the study did not fully explore how cultural, language, and migration-related factors influence MO in these groups. This realist review will build on the findings of the MEMORABLE study, by specifically focusing on older people from EMCs to better understand how, why, and under what circumstances MO works or fails to work for them in primary care.

Key questions guiding this review:

- How does the context, including culture, beliefs, and primary care-related factors, influence medicine optimisation?
- How do these contextual elements interact and drive the mechanisms to impact patient outcomes?
- What are the mechanisms that drive successful or unsuccessful medicine optimisation among older people in EMCs?

## Method

This protocol adheres to Preferred Reporting Items for Systematic Reviews and Meta-Analyses Protocols (PRISMA-P) guidelines (Additional file 1) [38]. It is also registered in the International Prospective Register of Systematic Reviews (PROSPERO) under the registration number CRD42023432204.

### Overview

A realist review aims to understand why interventions work or do not work, rather than summarising findings, by identifying underlying mechanisms and contexts [39–41]. This type of review adopts a systematic, iterative process, grounded on the principle of understanding ‘what works, for whom, in what circumstances, and why’. It strategically explores the complexity of interventions and their varied outcomes across diverse contexts [39, 40].

This approach is centred around context, mechanism, and outcome configurations (CMOCs) [39]. The ‘context’ relates to the setting against which the MO interventions occur [39]. Context includes everything from societal norms and family structures to the understanding of the healthcare policies and primary care practices that shape their experiences. ‘Mechanism’ refers to what prompts a change in the behaviour or circumstances of these patients [39], which could be the way information is communicated, and the trust between patient-practitioner. This interaction between context and mechanism is what makes healthcare services or interventions effective or ineffective in varying contexts [42]. ‘Outcomes’ extend beyond clinical results to include broader impacts such as improved understanding of medication, adherence to treatments, patient satisfaction, and overall quality of life [39].

This review will follow the structured and iterative five-step framework for realist synthesis proposed by Pawson et al., which includes developing initial programme theories (IPTs), searching for evidence, selecting and appraising studies, and synthesising the data [43]. Although the process has clear stages, it is flexible and adaptive, allowing for refinements based on emerging insights.

This review will follow both the conduct and reporting guidance set out by the Realist And Meta-narrative Evidence Syntheses: Evolving Standards (RAMESES) publication standards [44]. These standards will inform our realist logic of inquiry, including programme theory development, selection of evidence, data extraction using CMOCs, and retroductive reasoning, to ensure rigour, transparency, and explanatory depth in how we build and test theory.[44].

#### Step 1: develop the initial programme theories

Programme theories play a vital role in guiding realist reviews [45]. They are abstract representations that describe the components of interventions and lay down assumptions about how they lead to intended or observed outcomes. These theories explain the complex interaction between contexts, mechanisms, and outcomes, often represented as CMOCs [45].

For this study, we will develop ideas about IPTs from several sources, including empirical evidence (e.g. the MEMORABLE study, which highlighted challenges like limited patient involvement) [37], practitioners’ inputs, and formal theoretical frameworks. We will also use Bandura’s (1986) Social Cognitive Theory (SCT) as a theoretical framework [46]. SCT provides insights for better understanding the connection between individual behaviours, personal factors, and the social environment, which is particularly relevant in the context of MO among older people from EMCs [46]. While other theories, such as the Health Belief Model and Theory of Planned Behaviour, might provide some understanding of health decisions [47, 48], they lack the focus on social and observational learning [49]. Neverthless, we recognise that other theories may also become relevant as the review progresses and new data patterns emerge. In which case, we will engage with practitioners to review and prioritise a maximum of 4 IPTs to test against the literature.

#### Step 2: develop the search strategy

Secondary data from academic and grey literature was used to refine further the initial programme theory/theories. We will conduct an iterative literature search, with different search terms and combinations to find the most relevant data to support this process, NH will develop and refine the search terms in collaboration with IM and CB, and with input from an information specialist. A draft search strategy for Embase is provided in Additional file 2.

Before conducting the full multi-database search, a pilot search will be carried out using two databases (MEDLINE and Embase). This pilot phase will help us test and refine the draft strategy, ensuring that the selected search strings yield a manageable number of relevant results. Based on the pilot outcomes, we will adjust keywords and combinations accordingly in consultation with our information specialist.

The full search strategy will be designed to identify literature contributing to the development of CMOCs. The main databases to be searched include MEDLINE/PubMed, Embase, Scopus, Web of Science, the Cochrane Library, CINAHL, and PsycINFO. Grey literature will be sourced from ProQuest Dissertations & Theses Global, the King’s Fund Library, NHS Evidence, and NICE.

Additionally, we will use backward citation tracking (examining reference lists of included studies) and forward citation tracking (identifying newer studies that cite the included documents) [50]. Snowballing techniques will also be employed to locate further relevant papers that may add contextual or theoretical depth to the review [51].

The search strategy is built around the following elements:

- *Context:* older people (60 years and above) from EMCs with polypharmacy in primary care.
- *Intervention:* interventions aimed at optimising medicine use and the experiences of these older individuals, their family carers, and practitioners.
- *Mechanisms:* identified from the programme theories.
- *Outcomes:* quality of life, adherence, adverse events, disease symptoms, and patient satisfaction, as well as unexpected outcomes.

#### Steps 3 and 4: selection and appraisal of evidence

We will follow a systematic two-step process to select and appraise articles, utilising RAYYAN (a web-based application that facilitates the screening of titles and abstracts) [52]. The first step will start by screening titles and abstracts against inclusion and exclusion criteria (Table 1). A 20% random sample will be checked by a second reviewer (CB). Any disagreements will be resolved through discussion or, if necessary, by consulting with the rest of the team. If we find that there are frequent disagreements in the 20% sample, we will increase the number of articles that are double-screened and review our inclusion and exclusion criteria to make sure they are clear and consistent. For the second step, the documents which pass the initial screening will then undergo a full-text review to assess their relevance, rigour, and richness.

**Table 1:** Inclusion and exclusion criteria

**Inclusion criteria**	**Exclus**ion criteria
Aged 60 years and above, from ethnic minority communities Practitioners involved in their care Their Informal carers, who provide care or support in any capacity (e.g. family, friends)	Outside of primary care settings
Studies focusing on medicine optimisation and its interventions	Studies not focusing on medicine optimisation and its interventions
Qualitative, quantitative, and mixed methods study designs	Studies with no clear or relevant design
English language studies	Non-English language studies
Any study with relevance to the target population or condition	Studies with no relevance to the target population or condition

Although many population-level statistics and studies define older people as aged 65 and over, this review adopts a threshold of 60 years and above [53]. This reflects definitions used in several included studies and acknowledges that individuals from EMCs may experience earlier onset of age-related health conditions [54].

To align with the realist approach, included studies must contain data that allows for the identification and coding of context, mechanisms, and/or outcomes, even if these elements are not explicitly labelled as such [40, 44]. Also, we recognise that mechanisms may not always be explicitly labelled in the literature [42]. Therefore, in addition to theory-informed terms, we will include studies that contain process evaluations, implementation, and qualitative studies that may offer rich descriptions from which mechanisms can be inferred. Such documents will be identified during screening for relevance, richness, and rigour, not just by keyword hits [44].

We use ‘ethnic minority communities’ as a broad, inclusive term in our inclusion criteria, while our search strategy lists specific ethnic groups and related terms to ensure we capture studies using a wide range of descriptors.

We will also consider relevant grey literature, including policy documents, professional guidelines, reports, and opinion pieces, where they offer useful insights for theory development.

Polypharmacy will be pragmatically defined based on how it is reported in each included article. While we generally consider polypharmacy to mean the use of five or more medications, we will include studies using alternative definitions (e.g. ≥ 4 or ≥ 10 medications), provided they focus on the experiences or impacts of managing multiple medicines and contribute to programme theory development.

The full-text articles will be critically assessed and rated on a scale of one to five stars in RAYYAN, reflecting their relevance, richness, and rigour in contributing to the development of the programme theory.

### Relevance

Relevance will be assessed by asking the following questions of the articles:

(i) Do they provide insights or data into the challenges and considerations of MO for older people from EMCs dealing with polypharmacy in primary care?
(ii) Are they relevant to the development or refinement of Programme theories? [44].

We will use a relevance ranking system, used in other studies [55], with flexibility for studies outside the target age range (60 years and above) if they provide valuable insights:

- *High relevance (4–5 stars)*: direct insights into older people from EMCs’ medicine optimisation in primary care, specifically with polypharmacy. Studies including ages below 60 (e.g. 18–59) are considered if they offer significant, applicable insights.
- *Moderate relevance (3 stars):* related insights into medicine optimisation, potentially applicable EMCs. Must provide considerable information that is relevant to the olders’ specific challenges.
- *Low relevance (2 stars):* general discussions on medicine optimisation or polypharmacy are not focused on the target older demographic or significantly younger populations.
- *No relevance (1 star):* studies not aligning with the above criteria are excluded.

One-star ratings will indicate irrelevance, leading to exclusion, while two-star ratings will mark documents as uncertain, which will require a discussion with the second reviewer for potential re-evaluation. Three-star documents, although not directly contributing to the core theory, will provide useful background and therefore be included for context. Four-star and five-star documents will be identified as highly relevant and rich and will be integrated into the analysis, directly informing, and refining the programme theory.

### Rigour

Rigour is defined as the trustworthiness and credibility of the data [44]. When evaluating studies based on rigour, we will examine the study's design, methodology, data collection, and analysis techniques [56]. Documents that offer significant insights or potential contributions to theory development, even if not the most rigorously conducted, may still be included.

However, a particular focus will be given to studies that demonstrate a high degree of rigour. Richness.

Richness refers to the depth and detail with which studies explore and present their findings and implications, especially concerning peoples’ lived experiences, challenges, and outcomes [57]. Although structured tools such as the EMMIE framework have been proposed for assessing evidence in other fields (e.g. crime prevention) [58], we will follow a relevance-rigour-richness approach commonly adopted in health-focused realist reviews [44]. This approach allows for more flexibility and interpretive depth when analysing mixed and qualitative data relevant to MO.

#### Step 5: data extraction and synthesis

We will upload the full texts of the included papers into NVivo. This qualitative data analysis software tool will be used to help with coding relevant sections of the texts that relate to MO contexts, mechanisms, and their relationships to outcomes [59]. The characteristics of each document (such as study objectives, methods, participant demographics, and key findings) will be systematically extracted and organised into an Excel spreadsheet.

The coding process within NVivo will include inductive, deductive, and retroductive approaches. Inductive coding will allow themes to naturally emerge from the data, ensuring the analysis is grounded and responsive to the data itself [60]. Deductive coding involves applying pre-established codes based on the initial Programme theories, which maintain alignment with the theoretical framework used [60]. Retroductive coding will delve deeper, interpreting data of underlying causal mechanisms, thereby uncovering richer layers of understanding [61].

The data analysis will adopt a realist logic, which aims to make sense of the initial programme theories [44]. A series of critical questions will support this process to guide the analysis and synthesis of data, focusing on relevance, richness, and rigour. These questions will include interpreting the meaning of data in the context of the program theory, making judgments about partial or complete CMOCs based on the data, and understanding how these CMOCs relate to the overarching program theory. Also, interpretive cross-case comparisons will be employed to explain how and why specific outcomes occur, such as by examining differences in the levels of engagement in MO and cultural differences. Throughout this process, various forms of reasoning, including juxtaposition, reconciliation, adjudication, and data consolidation, will be employed [41]. The SCT will be revisited as one analytical lens to help explain emerging patterns within CMOCs, especially those involving behavioural and social influences. However, we remain open to incorporating other relevant theories if the data suggest different explanatory pathways in line with the iterative and theory-informed nature of realist analysis.

## Discussion

The realist review aims to understand how MO works/does not work for older people from EMCs with polypharmacy in primary care. This method will give a deep understanding of what works, for whom, and under which circumstances. Once we understand what is likely to work, we can start developing effective interventions.

Recent studies increasingly support the use of realist approaches in MO. For example, a review found that multidisciplinary medication reviews are most effective when supported by clear roles, continuity of care, and strong team communication [62]. Another review showed that person-centred deprescribing requires trust, shared understanding, and ongoing support [63].

A key strength of the realist approach is its ability to synthesise diverse forms of evidence, including qualitative, quantitative, and grey literature, and to produce explanatory insights about complex processes. This approach is also reflexive and theory-driven, enabling the development of context-sensitive recommendations that are more useful for policy and practice in real-world settings.

However, the approach also has limitations. Realist reviews rely on interpretive judgment, which introduces the potential for bias. The quality of findings depends on the richness and relevance of available data, which may vary across studies. Furthermore, identifying mechanisms can be challenging, particularly when they are not explicitly stated in primary studies.

A practical limitation in our review is the current technical issue preventing access to the British Library's EThOS system, which limits the inclusion of potentially insightful theses and dissertations. To mitigate this, we will use alternative platforms such as ProQuest Dissertations and Theses Global. Additionally, the review is limited to English language studies [64], which may introduce language bias and exclude valuable research published in other languages [65].

Finally, the findings will reflect the specific contexts and cultural settings of the studies reviewed. As such, the transferability of the findings beyond these contexts may be limited [66]. Nevertheless, by following the RAMESES framework and reporting standards, we aim to ensure methodological rigour, transparency, and reflexivity throughout the process [44].

Despite these challenges, this review is expected to make a valuable contribution by generating testable CMOCs that explain what works, for whom, and under what circumstances in MO for older people from EMCs. These insights can inform future research and support person-centred care in primary care settings.

We plan to disseminate our findings through peer-reviewed publications and presentations at relevant national and international conferences.

## Supplementary Information

Additional file 1: PRISMA-P 2015 Checklist.

Additional file 2: Embase.

## Data Availability

Not applicable.

## Abbreviations

CMOCs: Context Mechanism Outcome Configurations
EMCs: Ethnic Minority Communities
IPTs: The Initial Programme Theories
MO: Medicine Optimisation
RAMESES: Realist and Meta-narrative Evidence Syntheses: Evolving Standards
