# Supplementary material for: Understanding the challenges of medicine optimisation among older people (aged 60 years and above) from ethnic minority communities with polypharmacy in primary care: a realist review protocol
Source: Syst Rev. 2025 Aug 15;14:166. doi: 10.1186/s13643-025-02920-1 (PMC12355808; doi:10.1186/s13643-025-02920-1)
Supplement: Supplementary file 2 — Additional file 2: Embase. [file 13643_2025_2920_MOESM2_ESM.pdf]

Embase

Host: Ovid

Date range searched: 1974 to present.

Date searched: 30/01/2024.

Searcher: NH

Hits n: 269

1	(Medic* Management or medic* optimi?ation or drug utili?aton review or medic* reconcil* or medic* review or structured medic* review or deprescri*).m_titl.	12406
2	(Medic* adherence or Medic* compliance or patient satisfaction or "Inappropriate prescribing" or Overprescrib* or "Medication burden" or "adverse events").m_titl.	40928
3	(older or elderly or aging or "old age" or "late life" or "60 years and above" or Geriatric*).m_kw, ab, titl.	1976798
4	(Middle East* or Afric* or Asia* or Caribbean or "West Indies" or Bangladesh* or China* or India* or Somali* or Ethiopia* or Nigeria* or Kenya* or Uganda or Syria* or Pakistan* or ethnic minorit* or Black or Asian or "people of colour" or Race* or "mixed race" or "mixed racial" or "Black British" or "indian subcontinent" or Gyps* or "irish traveller" or "African Americans" or "Asian Americans" or Blacks or "Hispanic Americans" or Arabi* or Hindu* or Hindi or Muslim or Islam* or Tamil* or Lanka* or Urdu or Bengali* or Emigran* or Immigran* or Refugee* or migrant* or asylum seeker* or BAME or BME). m_kw, ab, titl.	2529523
5	(Polypharmacy or multipl* medic* or multipl* drug* or many medic* or "many drugs"). m_kw, ab, titl.	75407
6	("Primary care" or "Community health" or Family practi?e or General Practitioner or Pharmacy or GP or Family Medicine). m_kw, ab, titl.	609399
7	1 or 2 or 5	126360
8	3 and 4 and 6 and 7	269
